# Screening for Vulnerability in Older Cancer Patients: The ONCODAGE Prospective Multicenter Cohort Study

**DOI:** 10.1371/journal.pone.0115060

**Published:** 2014-12-11

**Authors:** Pierre Soubeyran, Carine Bellera, Jean Goyard, Damien Heitz, Hervé Curé, Hubert Rousselot, Gilles Albrand, Véronique Servent, Olivier Saint Jean, Isabelle van Praagh, Jean-Emmanuel Kurtz, Stéphane Périn, Jean-Luc Verhaeghe, Catherine Terret, Christophe Desauw, Véronique Girre, Cécile Mertens, Simone Mathoulin-Pélissier, Muriel Rainfray

**Affiliations:** 1 Department of Medical Oncology, Institut Bergonié, Comprehensive Cancer Centre, Bordeaux, France; 2 University of Bordeaux, Bordeaux, France; 3 Clinical and Epidemiological Research unit, Institut Bergonié, Comprehensive Cancer Centre, Bordeaux, France; 4 INSERM U897 (Institut national de la santé et de la recherche médicale), CIC1401 (Centre d′investigation clinique), Institut Bergonié, Bordeaux, France; 5 Oncogeriatric Coordination unit, Centre Jean Perrin, Clermont-Ferrand, France; 6 Oncology and Hematology unit, Centre Hospitalier Universitaire de Strasbourg - Hôpital de Hautepierre, Strasbourg, France; 7 Geriatric unit, Institut Jean Godinot, Reims, France; 8 Cancer Support unit, Institut de Cancérologie de Lorraine Alexis Vautrin, Vandoeuvre les Nancy, France; 9 Geriatric Evaluation and Management unit, Antoine Charial Hospital, Francheville, Lyon, France; 10 Breast Cancer unit, Centre Oscar Lambret, Lille, France; 11 Internal Medicine unit, Hôpital européen Georges-Pompidou, Paris, France; 12 Surgical Oncology unit, Institut de Cancérologie de Lorraine Alexis Vautrin, Vandoeuvre les Nancy, France; 13 Medical Oncology unit, Centre Léon Bérard, Lyon, France; 14 Senology unit, Hôpital Saint Vincent de Paul, Université Catholique de Lille, Lille, France; 15 Oncology and Haematology unit, Centre Hospitalier Départemental, La Roche sur Yon, France; 16 Department of Clinical Gerontology, Centre Hospitalier Universitaire de Bordeaux, Bordeaux, France; Van Andel Institute, United States of America

## Abstract

**Background:**

Geriatric Assessment is an appropriate method for identifying older cancer patients at risk of life-threatening events during therapy. Yet, it is underused in practice, mainly because it is time- and resource-consuming. This study aims to identify the best screening tool to identify older cancer patients requiring geriatric assessment by comparing the performance of two short assessment tools the G8 and the Vulnerable Elders Survey (VES-13).

**Patients and Methods:**

The diagnostic accuracy of the G8 and the (VES-13) were evaluated in a prospective cohort study of 1674 cancer patients accrued before treatment in 23 health care facilities. 1435 were eligible and evaluable. Outcome measures were multidimensional geriatric assessment (MGA), sensitivity (primary), specificity, negative and positive predictive values and likelihood ratios of the G8 and VES-13, and predictive factors of 1-year survival rate.

**Results:**

Patient median age was 78.2 years (70-98) with a majority of females (69.8%), various types of cancer including 53.9% breast, and 75.8% Performance Status 0-1. Impaired MGA, G8, and VES-13 were 80.2%, 68.4%, and 60.2%, respectively. Mean time to complete G8 or VES-13 was about five minutes. Reproducibility of the two questionnaires was good. G8 appeared more sensitive (76.5% versus 68.7%, *P* =  0.0046) whereas VES-13 was more specific (74.3% versus 64.4%, *P*<0.0001). Abnormal G8 score (HR = 2.72), advanced stage (HR = 3.30), male sex (HR = 2.69) and poor Performance Status (HR = 3.28) were independent prognostic factors of 1-year survival.

**Conclusion:**

With good sensitivity and independent prognostic value on 1-year survival, the G8 questionnaire is currently one of the best screening tools available to identify older cancer patients requiring geriatric assessment, and we believe it should be implemented broadly in daily practice. Continuous research efforts should be pursued to refine the selection process of older cancer patients before potentially life-threatening therapy.

## Introduction

Cancer occurs predominantly in the older population, yet patients over 60 years are significantly under-represented in clinical trials in oncology [1], [2]. Consequently, oncologists are confronted with the paucity of clear therapeutic directives, and older patients are often offered reduced treatments and face worse outcomes [3], including an increased risk of toxicity or even early death [4]. With growing numbers of older cancer patients, and considerable heterogeneity among them, effective tools are required for oncologists to better define the trade-off between treatment benefits and toxicity risk.

Several recent reports have strongly suggested that different components of comprehensive (CGA) or multidimensional geriatric assessment (MGA) can be useful in oncology to predict early death [4], functional decline [5], toxicity [6], [7] and ultimately survival [8]–[10], and to adapt cancer treatment [11]. However, despite recommendations from the International Society of Geriatric Oncology (SIOG) [12], CGA is still underused in practice. The likely reason is that it is time- and resource-consuming, which makes it unaffordable for community and small cancer hospitals. Furthermore, true CGA (in contrast to MGA which involves the administration of a range of assessments) is conducted by an experienced geriatrician who interprets and can act upon the MGA results, and geriatricians are rarely available in most cancer treatment structures. This has made the development of shortened instruments essential [13], [14]. To be acceptable for the whole community, such instruments should be performed quickly (less than 10 min) by a nurse or physician trained for the tool completion, but not necessarily trained in geriatrics.

In response to a French National Cancer Institute (INCa) call for proposal, and following escalating appeals for validated geriatric screening tools [15]–[17], we developed the G8 screening tool to identify older cancer patients requiring geriatric assessment. The G8 tool originated from a regional multicenter prospective cohort of 364 cancer patients treated by first-line chemotherapy [18], [19]. The reference test (or “gold-standard”) was defined as at least one abnormal geriatric assessment test among seven. Preliminary results indicated 85% sensitivity and 65% specificity, which was promising given the priority of a screening test for maximum sensitivity to minimize the number of patients not detected.

We subsequently launched the national ONCODAGE multicenter study to validate the G8 tool prospectively. The primary objective was to validate the G8 instrument as a screening tool to identify older cancer patients (>70 years) requiring geriatric assessment by comparing with a reference test of MGA. Secondary objectives included assessing the diagnostic accuracy of G8 in specific sub-populations, the diagnostic accuracy of VES-13 and comparing it to that of G8, the within-patient reproducibility of both tests, and the prognostic value of both tests in terms of 1-year survival. Additional exploratory analyses included the assessment of the diagnostic accuracy of G8 and VES-13 using a modified reference test (at least two MGA tests with abnormal scores), and sensitivity analyses to assess the impact of missing questionnaires in the definition of the reference test.

## Methods

### Patients

We recruited patients from 23 health care facilities, including the 15 INCa-accredited Regional Coordination Units for Geriatric Oncology. Patients eligible were older than 70 years and were included either before any first-line treatment, or between any two steps of a pre-defined first-line treatment sequence (chemotherapy, endocrine therapy, targeted treatment, surgery or radiotherapy) for various types of histologically-confirmed cancer (colon, lung, upper aero digestive tract (UAT)/head and neck, breast, prostate, and non-Hodgkin's lymphomas (NHL)). Patients with known central nervous system metastases were excluded. Patients were informed of the study and provided their signed informed consent prior to enrollment and G8/MGA assessment. The protocol was approved by the regional ethics committee (Comité de Protection des Personnes Sud-Ouest et Outre Mer III), and was conducted in accordance with the Declaration of Helsinki, Good Clinical Practices (Trial registration: NCT00963911).

### Test methods

#### The G8 index test

At the first visit after enrollment, patients received a full clinical examination and completed the G8 test with a nurse, a clinical research assistant (CRA), or a physician. The G8 consists of eight items: patient age (>85, 80-85, <80), and seven items from the original 18-item MNA (appetite changes, weight loss, mobility, neuropsychological problems, body mass index, medication, and self-rated health). The total score ranges from 0 to 17, with lower scores indicating a higher risk of impairments.[18] The cut-off value for an ‘impaired’ reference test score was ≤14 and the time taken to complete the test was recorded. The G8 questionnaire is provided in S1 Appendix.

#### The VES-13 questionnaire

VES-13 is a self-administered questionnaire that was completed during the first visit after enrollment. For three pre-identified centers, patients also filled in the questionnaire at the following geriatric visit. VES-13 consisted of four groups of questions: age, self-perceived health, difficulties to perform six specific activities, and difficulties to perform daily living tasks due to health concerns. The score ranged from 0 to 10 and a score ≥3 was considered to show impairment.

#### Multidimensional geriatric assessment (MGA) reference test

Patients underwent a geriatric evaluation in the month following the completion of G8 and VES-13 (+/- seven days) before treatment began. The nurse completed six of the seven instruments of the MGA as already described [18] (MNA, Timed Get up and Go (TUG), Activities of Daily Living (ADL), Instrumental ADL (IADL), Mini Mental State Examination (MMSE), and Geriatric Depression Scale (GDS-15)), and the geriatrician rated comorbidity on the Cumulative Illness Rating Scale (CIRS-G), recorded the time required for the consultation, identified patients who needed personalized geriatric interventions, and, if necessary, proposed further geriatric evaluation (outside of the scope of this study). G8 results were blinded to both the geriatrician and the nurse.

Abnormal scores for each instrument were established according to the following published cut-offs [18]: at least one Grade ≥3 comorbidity on the CIRS-G [20] (excluding the cancer being treated); ADL≤5, IADL≤7 across genders, MNA≤23.5, MMSE≤23/30, GDS15≥6, and TUG>20 seconds. Based on preliminary analyses [18], we considered the reference test to be ‘impaired’ if scores on the seven instruments were available and one or more of them was abnormal, or if the score on one or more instruments could not be calculated due to one or more missing item or unavailable instrument.

The reference test was defined as normal if scores for the seven instruments were available and normal. In a subsequent exploratory analysis, the reference test was modified and we considered the reference test to be ‘impaired’ if the seven instruments were available with two or more abnormal scores, or if the score for two or more instruments could not be calculated due to one or more missing items or unavailable instruments.

### Statistical methods

We defined the following populations: the included population, the eligible population, and the eligible and evaluable population. The included population corresponded to all patients included, regardless of eligibility and availability of G8 and MGA results. The eligible population included all patients who did not violate any eligibility criteria. The eligible and evaluable population for diagnostic accuracy assessment was defined as all eligible patients, for whom G8 as well as at least one instrument of MGA were available and were administered less than one month apart (± one week).

Diagnostic accuracy was measured by the classification probabilities (sensitivity and specificity), positive and negative predictive values (PPV and NPV), and positive and negative diagnostic likelihood ratios (+/-DLR). The McNemar test was applied to compare the sensitivity of G8 and VES-13, as well as their specificity.

The required sample size was estimated based on our preliminary work [18]. Assuming 90% sensitivity for the G8 tool [18], we calculated that the enrollment of 750 patients with at least one abnormal MGA instrument would allow us to estimate sensitivity with sufficient precision (2.4%) and to obtain 95%CIs between 87.6% and 92.4%. Based on an estimated 50% of patients with at least one abnormal MGA instrument [21], 1500 eligible and evaluable patients were required. Assuming 10% ineligibility, this involved recruitment of 1650 patients.

The study population was described in terms of clinical and demographic characteristics with counts and percentages for qualitative variables and summary statistics (mean and variance where appropriate; percentiles otherwise) for quantitative variables. Sensitivity, specificity, PPV, NPV, +/-DLR and area under the ROC curve were estimated with their (two-sided) 95%CI.

Reproducibility analysis was based on estimation of the Kappa agreement statistics for dichotomous data (normal *v* abnormal score) [22].

Reproducibility of G8 was assessed by comparing the score on the actual G8 with the scores extracted from the corresponding seven questions of MNA completed during the MGA for all patients. Reproducibility of VES-13 was assessed based on a subgroup of patients included in three pre-identified centers who completed the questionnaire on two occasions. A priori sample size estimation suggested that enrollment of at least 180 subjects would ensure sufficient precision to estimate the reproducibility of VES-13 in this subgroup.

The prognostic value of the screening tools was assessed by analyzing one-year overall survivals using a Cox proportional hazards model. Candidate prognostic factors included age, sex, ECOG PS (Eastern Cooperative Oncology Group performance status), stage (metastatic *v* non-metastatic), and the G8 score. Significant factors at the univariate stage (p<5%) were subsequently included in a multivariate model. The final model was based on a manual stepwise backward selection approach with statistical significance set at 5%. An exploratory model was also calculated examining the prognostic value of the reference test MGA score. Hazard ratios (HR) are reported with 95%CIs.

Results are presented according to the STARD guidelines [23] for reporting of studies of diagnostic accuracy, and the study protocol is available in S2 Appendix.

## Results

### Enrollments

**Figure 1:**
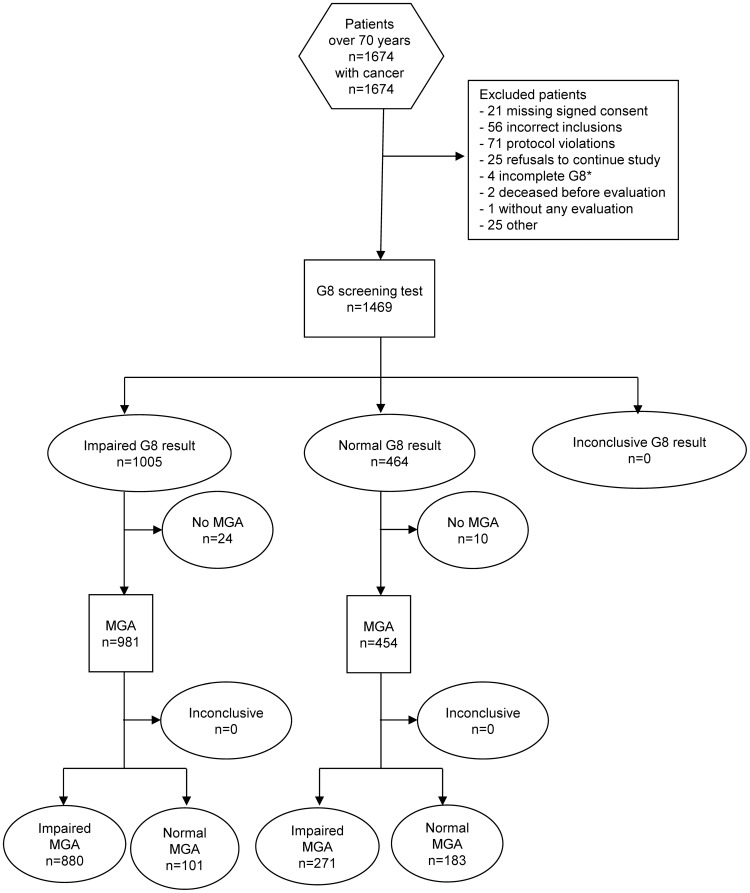
STARD flow diagram for patient enrollments and exclusions in the ONCODAGE G8 study. Footnote: *In total, ten G8 were incomplete, but six ‘abnormal’ scores were able to be imputed from the incomplete assessments.

Between August 2008 and March 2010, 1674 patients were included in the ONCODAGE study. Initial exclusion of 77 ineligible patients left 1597 patients (eligible population). A further 162 patients were excluded from analyses due to protocol violations, participation withdrawals, missing G8 or MGA (Fig. 1). Delay between G8 and MGA exceeded 37 days in 15 cases, and G8 was inadequately completed by three patients. The final eligible and evaluable population for the principal analyses consisted of 1435 patients with a median age of 78 years and of whom 69.5% were females (Table 1). Patients were mostly seen in first consultations by a medical oncologist (45.6%), surgeons (21.7%), radiotherapists (13.5%), or other cancer specialists (19.0%).

**Table 1:** Patient and tumor characteristics in the ONCODAGE project.

	Eligible	Eligible and evaluable
	n = 1597	n = 1435
**Median age** (range) (years)	78 (70 to 98)	78 (70 to 98)
70–74	493 (30.9)	444 (30.9)
75–79	481 (30.1)	433 (30.2)
80–84	392 (24.5)	354 (24.7)
85+	231 (14.5)	204 (14.2)
**ECOG PS** *		
0	608 (38.1)	549 (38.2)
1	518 (32.4)	476 (33.2)
2	225 (14.1)	199 (13.9)
3	93 (5.8)	82 (5.7)
4	48 (3.0)	46 (3.2)
Missing	105 (6.6)	83 (5.8)
**Sex**		
Male	487 (30.5)	434 (30.2)
**Tumor site**		
Breast	852 (53.4)	774(53.9)
Colon-rectum	229 (14.3)	204 (14.2)
Lung	168 (10.5)	149 (10.4)
Prostate	141 (8.8)	122 (8.5)
Non-Hodgkin's Lymphoma	120 (7.5)	112 (7.8)
Upper Aero Digestive Tract/Head and Neck	87 (5.5)	74 (5.2)
**Disease stage** **		
Non-metastatic (M0)	895 (56.0)	810 (56.4)
Non-metastatic (MX)	269 (16.8)	246 (17.1)
Metastatic (M1)	284 (17.8)	249 (17.3)
Missing	149 (9.3)	130 (9.1)
**Treatment performed before evaluation** ***	561 (35.1)	497 (34.7)
Surgery	431 (27.0)	384 (26.8)
Endocrine therapy	108 (6.8)	91 (6.3)
Chemotherapy	29 (1.8)	25 (1.7)
Radiotherapy	26 (1.6)	22 (1.5)
Radio-chemotherapy	6 (0.4)	6 (0.4)
Targeted therapy	4 (0.3)	4 (0.3)
Watchful waiting	4 (0.3)	4 (0.3)
**Treatment planned after evaluation** ***		
Radiotherapy	662 (41.5)	596 (41.6)
Chemotherapy	559 (35.1)	510 (35.6)
Surgery	518 (32.5)	476 (33.2)
Endocrine therapy	479 (30.1)	433 (30.2)
Targeted therapy	94 (5.9)	83 (5.8)
Radiochemotherapy	72 (4.5)	62 (4.3)
Watchful waiting	23 (1.4)	20 (1.4)

*ECOG PS = eastern cooperative oncology group performance status.

**Recommendations of the Cancer Care Ontario Practice Guidelines Initiative were implemented.

***As part of the first-line treatment initially planned. More than one treatment possible.

Values are numbers (percentages) unless stated otherwise.

After the first 779 enrollments, we convened an international independent data monitoring committee to examine recruitment across different tumor sites and discuss initial statistical hypotheses. No modification was proposed by the committee of experts.

### The multidimensional geriatric assessment reference standard results

On average, it took one hour to complete the MGA overall (67.5 minutes, +/-24.6; range 10 minutes to 3 hours) and it was completed in less than one and a half hours for 75% of patients. Almost all (91.6%) patients completed the seven instruments entirely. Rates of completion varied across instruments from 97.0% for the GDS15 to 99.8% for the ADL and MNA. The proportion of patients with abnormal scores varied from 15.3% for the ADL to 47.8% for the IADL (Table 2). Similar results were found for the eligible population (n = 1597).

**Table 2:** Percentages of normal and abnormal scores on the reference standard Multidimensional Geriatric Assessment (MGA) instruments for eligible and evaluable patients (n = 1435) and for eligible patients.

	Eligible and evaluable (n = 1435)				Eligible (n = 1597)			
	Normal		Abnormal**		Normal		Abnormal	
MGA instrument*	n	%	n	%	n	%	N	%
ADL	1216	84.7	219	15.3	1283	84.8	230	15.2
IADL	749	52.2	686	47.8	788	53.3	691	46.7
GDS15	974	67.9	461	32.1	1027	70.2	435	29.8
MMSE	1143	79.6	292	20.3	1203	80.0	300	20.0
MNA	808	56.3	627	43.7	851	56.7	649	43.3
CIRS-G	833	58.0	602	41.9	879	59.4	601	40.6
TGUG	1105	77.0	330	23.0	1162	77.3	341	22.7

*ADL = activities of daily living; IADL = instrumental activities of daily living; GDS = geriatric depression score; MMSE = mini-mental state examination; MNA = mini nutritional assessment; CIRS-G = comorbidities rating scale – geriatrics; TGUG =  Timed Get Up and Go.

** Abnormal scores were defined per instrument as (for complete instruments): ADL ≤ 5/6, IADL ≤ 7/8, GDS15 ≥ 6/15, MMSE ≤ 23/30, MNA ≤ 23.5/30, CIRS-G presence of at least one comorbidity (excluding the cancer being treated), and TGUG> 20 seconds. Incomplete or unavailable instruments were considered abnormal.

Overall, 1151 (80.2%) eligible and evaluable patients were considered to have an impaired reference test. This was determined for the large majority of patients (1031, 89.6%) who had an abnormal score on one or more of any of the seven available instruments. For the remaining 120 patients (10.4%), the score from at least one instrument was missing and could not be determined. For 85 of these patients, although at least one score was missing, the score on one of the remaining instruments was abnormal so their reference test was considered impaired. For the remaining 35 patients with only five or six available scores, all available scores were normal. Their reference test was considered to be impaired for the purposes of the main analyses (see further discussion and analyses in results).

Of the 1031 patients overall with altered scores, 306 (29.7%) had an altered score on one instrument, 236 (22.9%) on two, 173 (16.8%) on three, 132 (12.8%) on four, 94 (9.1%) on five, 73 (7.1%) on six and 17 (1.6%) on seven.

Proportions of subjects with at least one impaired score varied across disease stage (93.0% for metastatic patients (M1) *v* 75.1% for non-metastatic (M0)) and across tumor site (73.0% prostate, 74.2% breast, 86.6% NHL, and 91 to 92% for colon rectum, lung and UAT/head and neck). At least one geriatric intervention was proposed by the geriatrician at the end of MGA in 72.2% of the cases and between one and four per patient in 64.3% of the population. The most frequently proposed interventions were nutritional support (524 patients, 37.0%), home assistance (499 patients, 35.2%), standard treatment adaptation (345 patients, 24.3%), psychological support (258 patients, 18.3%) and physiotherapy (231 patients, 16.3%).

### Validation of the G8 test

In the eligible and evaluable population (n = 1435), G8 was mostly administered by a nurse or CRA (87%) and less frequently by a physician (12.9%). It took an average of 4.4 minutes to complete (+/−2.8, range: 1–60 minutes) with 98.7% completed in ten minutes or less. The final G8 scores ranged from 1.5 to 17, with 68.4% of patients showing impaired scores (≤14). The proportions of patients with impaired G8 scores varied according to disease stage (85.6% for M1 and 63.0% for M0) and tumor site (36.9% prostate, 62.9% breast, 70.5% NHL, and 85–88% for colon-rectum, lung and UAT/head and neck).

**Figure 2:**
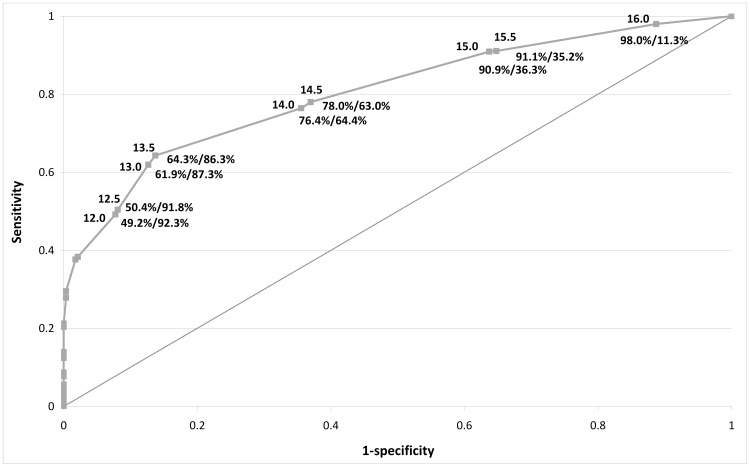
ROC curve for G8 test *v* MGA reference standard in the ONCODAGE study*. Footnote: * For each point on the curve the G8 threshold (above line) and the sensitivity and specificity (below line) are indicated, e.g. score of 14, sensitivity: 76.4%, specificity: 64.4%. Abbreviations: ROC =  Receiver Operating Characteristics; MGA =  Multidimensional Geriatric Assessment.

The diagnostic accuracy of G8 is outlined in Table 3. G8 sensitivity was 76.5% and specificity 64.4%. The AUC compared to the reference standard MGA was 0.804, 95%CI 0.78 to 0.83 (Fig. 2).

**Table 3:** Diagnostic accuracy of G8 and Vulnerable Elders Survey (VES-13)* screening tools for identifying older patients who could benefit from Multidimensional Geriatric Assessment (MGA) for eligible and evaluable population (n = 1435).

	Reference test *	Modified reference test *
	Estimation (95% CI)	95% CI
**G8**		
Sensitivity	76.5% (73.9 to 78.9)	86.5% (83.9 to 88.7)
Specificity	64.4% (58.6 to 70.0)	55.3% (51.3 to 59.3)
False negative rate	23.5% (21.1 to 26.1)	13.5% (11.2 to 16.1)
False positive rate	35.6% (30.0 to 41.4)	44.7% (40.7 to 48.7)
Positive Predictive Value	89.7% (87.6 to 91.5)	71.7% (68.7 to 74.5)
Negative Predictive Value	40.3% (35.8 to 45.0)	75.8% (71.6 to 79.6)
Positive Likelihood Ratio	2.15 (1.83 to 2.52)	1.93 (1.77 to 2.12)
Negative Likelihood Ratio	0.36 (0.32 to 0.42)	0.24 (0.20 to 0.30)
**VES13** **		
Sensitivity	68.7% (66.0 to 71.4)	78.5% (75.5 to 81.2)
Specificity	74.3% (68.8 to 79.3)	63.7% (59.7 to 67.4)
False negative rate	31.3% (28.6 to 34.0)	21.5% (18.7 to 24.5)
False positive rate	25.7% (20.7 to 31.2)	36.3% (32.5 to 40.2)
Positive Predictive Value	91.6% (89.5 to 93.3)	73.8% (70.8 to 76.7)
Negative Predictive Value	37.0% (33.0 to 41.1)	69.3% (65.4 to 73.1)
Positive Likelihood Ratio	2.67 (2.19 to 3.3)	2.16 (1.93 to 2.41)
Negative Likelihood Ratio	0.42 (0.38 to 0.5)	0.34 (0.29 to 0.39)

*The reference test is defined as one or more abnormal MGA tests; modified reference test is defined as 2 or more abnormal MGA tests.

**Vulnerable Elders survey 13 (French version) [44].

G8 was abnormal in 94.4% abnormal MNA (592/627), 93.6% abnormal ADL (205/219), 91.3% abnormal TGUG (293/321), 84.8% abnormal GDS15 (391/461), 84.5% abnormal IADL (580/686), 80.5% abnormal MMSE (235/292) and 77.4% abnormal CIRS-G (466/602). Overall, 136 patients with Grade 3-4 comorbidities had normal G8 scores; this included mainly patients with one severe comorbidity (58.1%) the most prevalent being vascular (49.3%), cardiac (15.4%), respiratory (14%) and metabolic (11.8%).

Among false negative results, 53.1% had only one abnormal MGA questionnaire (median 1; range 1 to 6) as compared to 18.4% in the true positive group (median 3; range 1 to 7).

### Secondary analyses

#### G8 diagnostic accuracy by subgroups

In terms of disease stage, sensitivity and PPV were superior for M1 patients than for M0 patients, whereas the specificity and NPV were better for M0 patients (Sensitivity  =  87.6% (M1) *v* 71.3% (M0); Specificity  =  43.8% (M1) *v* 65.7% (M0); PPV  =  95.8% (M1) *v* 86.3% (M0); NPV  =  19.4% (M1) *v* 43.0% (M0)) (Table 4). Sensitivity and specificity varied significantly according to tumor site: the sensitivity for prostate cancer patients was 46.1%, 72.3% for breast cancer patients and 94.1% for UAT/head and neck patients; and specificity ranged from 33.3% for lung cancer patients to 87.9% for prostate cancer patients (Table 4).

**Table 4:** Secondary analyses of diagnostic accuracy of G8 according to subgroups (n = 1435).

	Sensitivity	Specificity	PPV*	NPV*
**Non-Metastatic (n)**	609	201	503	307
Estimation	71.3	65.7	86.3	43.0
95%CI – lower bound	67.5	58.7	83.0	37.4
95%CI – upper bound	74.8	72.2	89.2	48.7
**Metastatic (n)**	233	16	213	36
Estimation	87.6	43.8	95.8	19.4
95%CI – lower bound	82.6	19.8	92.1	8.2
95%CI – upper bound	91.5	70.1	98.1	36.0
**Stage not available (n)**	309	67	265	111
Estimation	78.3	65.7	91.3	39.6
95%CI – lower bound	73.3	53.1	87.3	30.5
95%CI – upper bound	82.8	76.9	94.4	49.4
**Breast (n)**	574	200	487	287
Estimation	72.3	64.0	85.2	44.6
95%CI – lower bound	68.4	56.9	81.8	38.8
95%CI – upper bound	75.9	70.7	88.3	50.6
**Colon-rectum (n)**	186	18	174	30
Estimation	87.6	38.9	93.7	23.3
95%CI – lower bound	82.0	17.3	89.0	9.9
95%CI – upper bound	92.0	64.3	96.8	42.3
**Lung (n)**	137	12	131	18
Estimation	89.8	33.3	93.9	22.2
95%CI – lower bound	83.5	9.9	88.3	6.4
95%CI – upper bound	94.3	65.1	97.3	47.6
**Prostate (n)**	89	33	45	77
Estimation	46.1	87.9	91.1	37.7
95%CI – lower bound	35.4	71.8	78.8	26.9
95%CI – upper bound	57.0	96.6	97.5	49.4
**NHL**† **(n)**	97	15	79	33
Estimation	76.3	66.7	93.7	30.3
95%CI – lower bound	66.6	38.4	85.8	15.6
95%CI – upper bound	84.3	88.2	97.9	48.7
UAT**/Head and Neck (n)	68	6	65	9
Estimation	94.1	83.3	98.5	55.6
95%CI – lower bound	85.6	35.9	91.7	21.2
95%CI – upper bound	98.4	99.6	100.0	86.3
**Previous treatment NO** ^§^ **(n)**	771	166	668	269
Estimation	78.1	60.2	90.1	37.2
95%CI – lower bound	75.0	52.4	87.6	31.4
95%CI – upper bound	80.9	67.7	92.3	43.2
**Previous treatment YES** ^§^ **(n)**	380	117	313	184
Estimation	73.2	70.1	88.8	44.6
95%CI – lower bound	68.4	60.9	84.8	37.2
95%CI – upper bound	77.5	78.2	92.1	52.1

*Positive (PPV) and Negative (NPV) predictive values; † Non-Hodgkin's Lymphoma; ** Upper Aero Digestive Tract; ^§^Treatment in the last three months.

A subgroup analysis explored the influence of the presence or absence of at least one treatment in the last three months. G8 sensitivity or specificity did not appear to be particularly affected by this factor.

#### Diagnostic accuracy of VES-13

In the eligible and evaluable population (n = 1435), VES-13 was mainly administered with the assistance of a nurse or CRA (78.7%), with 12.1% being completed by the patient alone, and 8.8% with the assistance of the physician. On average, it took 5.7 minutes to complete (+/−3.2, range1–30 minutes), with 98% completed in less than 10 minutes.

VES-13 showed impaired scores in 60.2% of patients. Sensitivity and specificity were 68.7% and 74.3%, respectively (Table 3). The AUC for VES-13 compared to MGA was 0.79, 95%CI 0.77 to 0.82. Sensitivity and PPV were higher for M1 rather than for M0 patients (Sensitivity = 75.1% (M1) *v* 65.4% (M0); Specificity = 43.8% (M1) *v* 75.6% (M0); PPV = 95.1% (M1) *v* 89.0% (M0); NPV = 10.8% (M1) *v* 41.9% (M0)).

VES-13 was abnormal in 94.1% abnormal ADL (206/219), 87.5% abnormal TGUG (281/321), 81.5% abnormal IADL (559/686), 80.9% abnormal GDS15 (373/461), 77.5% abnormal MNA (486/627), 75.3% abnormal MMSE (220/292) and 72.4% abnormal CIRS-G (436/602). Overall, VES-13 was normal in 166 patients with Grade 3–4 comorbidities, which included mainly patients with one severe comorbidity (51.2%), the most prevalent being vascular (45.2%), cardiac (21.7%), respiratory (14.5%) and psychiatric (12.7%) comorbidities.

Among false negative results, 45.3% had only one abnormal MGA questionnaire (median 1; range 1 to 6) as compared to 18.1% in the true positive group (median 3; range 1 to 7).

#### Comparison of the G8 and VES-13 diagnostic accuracy

The G8 test had a better sensitivity than VES-13 (McNemar test, p =  0.005) at the expense of a lower specificity (McNemar test, p<0.0001).

#### G8 and VES-13 reproducibility

Reproducibility for both G8 (n = 1429) and VES-13 (n = 251) was good: Kappa = 0.65, (95%CI 0.61 to 0.70), and Kappa = 0.64, (95%CI 0.54 to 0.73), respectively. When investigating the items of each instrument, the Kappa coefficient varied from excellent for objective criteria such as age (G8: Kappa = 0.96, 95%CI 0.95 to 0.98; VES-13: Kappa = 0.98, 95%CI 0.94 to 1.0) to poor for subjective criteria such as self-rated health (G8: Kappa = 0.38; VES-13: Kappa = 0.42).

#### One-year survival of G8 and VES-13

Among the 1435 patients eligible and evaluable, information on vital status was available for 1365 patients with a median follow-up of 377 days (95%CI 373 to 381). At the univariate level, all candidate prognostic factors were statistically significant (Table 5). In the final model, factors independently associated with poorer survival included male sex (HR 2.69, p<0.0001), 2–4 ECOG status (HR 3.28, p<0.0001), metastatic disease (HR 3.30, p<0.0001), and abnormal G8 score (HR 2.72, p<0.0001) (Table 5).

**Table 5:** Factors associated with one-year survival (univariate and multivariate models).

	Univariate analysis (1365 patients)		Multivariate analysis (1167 patients)	
	Hazard ratio (95% CI*)	P value	Hazard ratio (95% CI*)	P value
**Age**				
70 – 74	Reference	0.0024	Reference	0.5582
75 - 79	1.12 (0.78 to 1.59)		0.85 (0.57 to 1.25)	
80 - 84	1.49 (1.04 to 2.12)		0.88 (0.60 to 1.30)	
85 and over	1.95 (1.33 to 2.88)		0.72 (0.45 to 1.14)	
**Sex**				
Female	Reference	<0.0001	Reference	<0.0001
Male	3.09 (2.40 to 3.99)		2.69 (2.02 to 3.58)	
**ECOG PS** *				
0-1	Reference	<0.0001	Reference	<0.0001
2-4	5.30 (4.07 to 6.90)		3.28 (2.41 to 4.46)	
**Stage**				
Non-metastatic	Reference	<0.0001	Reference	<0.0001
Mx**	1.09 (0.69 to 1.69)		1.14 (0.72 to 1.79)	
Metastatic	5.67 (4.23 to 7.60)		3.30 (2.42 to 4.50)	
**G8**				
Normal	Reference	<0.0001	Reference	<0.0001
Abnormal	4.72 (3.07 to 7.26)		2.72 (1.66 to 4.47)	

*ECOG PS =  Eastern Cooperative Oncology Group Performance Status.

**Mx  =  Unknown.

In a model including all significant candidate prognostic factors and MGA result, abnormal MGA was found to be significantly associated with poorer survival at one year (HR 2.96, 95%CI 1.50–5.85, p = 0.0018).

### Exploratory analyses

#### Diagnostic accuracy of G8 and VES-13 with the modified standard (two abnormal MGA tests)

Using a modified definition of the reference test (two abnormal MGA tests), 56.7% of patients (813 cases) were considered abnormal. G8 sensitivity was 86.5% and specificity 55.3% (Table 3). The AUC was 0.82, 95%CI 0.80 to 0.84. Sensitivity and specificity of VES-13 with this modified reference test were 78.5% and 63.7%, respectively (Table 3).

#### Sensitivity analysis to assess the impact of missing questionnaires of MGA

For 35 out of 1435 patients, scores for at least five instruments were available and normal. However, MGA was considered abnormal due to the one or two not fully completed questionnaires despite normality on all other available instruments. To specifically account for these patients with missing questionnaires, we investigated two modified definitions of the reference test. First, if two or less questionnaires were missing but all other MGA instruments had normal scores, then the reference test was considered normal, otherwise the definition of our primary reference test prevailed. Second, the reference test was considered normal as long as all completed questionnaires had a normal score (regardless of the number of missing questionnaires).

Using these new definitions of the reference test did not cause changes in the diagnostic performances of G8 (similar values for sensitivity, specificity, PPV, NPV and likelihood ratios, data not shown), so the primary definition with these 35 patients considered as abnormal reference tests was maintained.

## Discussion

This is the first study designed to determine the diagnostic accuracy of the G8 questionnaire and, by far, the largest prospective cohort available to validate a screening tool in geriatric oncology with previously published studies including 41–419 patients [24]–[32]. The G8 test proved to be convenient, easy and quick to administer. It was generally completed in less than five minutes and was mostly administered by a nurse with no specific expertise in geriatrics. So far, the G8 tool exists only in French, but can easily be applied in other languages using the official English MNA translation (Appendix S1), or one of the 22 other official translations.

The proportion of G8 impaired scores was 68.4%. In the validation against the reference test (0 *v* ≥1 abnormal MGA tests − 80% of the population), sensitivity, of foremost importance for a screening tool, was good at 76.5% and specificity was satisfactory at 64.4%. With the modified reference test (<2 *v* ≥2 abnormal MGA tests − 56.7% of the population), sensitivity of G8 was improved to 86.5% while specificity was reduced to 55.3%.

The population of the study was homogeneously defined including only first-line cancer treatment patients. As it has been developed in a large number of investigating centers in France, including community hospitals, and as it can be equally administered by a nurse or a physician, the G8 questionnaire can be smoothly implemented in daily practice. However, the large representation of breast cancer patients included in our study (over 50%) needs to be considered when generalizing the results. Consequently, we provide diagnostic accuracy estimations per tumor location in the subgroup analyses. There was a significant proportion of patients (9.1%) with missing metastatic status for whom physicians decided not to perform usual pre-treatment work-up because of low risk of metastasis and age.

Our target population is clearly identified with simple, quantitative criteria: at least one or two abnormal questionnaires among seven consensus geriatric tools. However, while MGA appears the best available and most reproducible instrument to identify it, this target population (reference test) has no unbiased definition. Two different thresholds considering one [18], [24], [27], [29] or two [18], [26], [30]–[33] abnormal questionnaires have been proposed in the literature with different sets of questionnaires that more or less cover geriatric domains [34]. So far, no objective arguments have been raised that enable us to choose the best threshold [35]. Selecting patients with at least one abnormal questionnaire (80% of patients in our series, 66% to 94% in other published studies) [18], [24], [27], [29] reduces the risk of missing unfit patients but also limits the validity of the screening procedure. With two abnormal tests as the threshold, the target population is smaller (56.7% in our series vs. 43% to 76% in the literature) [18], [26], [30]–[33], which may enable us to concentrate our efforts on the most vulnerable patients.

While considering sensitivity as the most important criteria (to limit false negative case occurrences), we believe G8 to be the best available tool, although VES-13 remains a good alternative with lower sensitivity but higher specificity. In this study we assessed the performance of VES-13, which is currently the most widely-used screening tool for older cancer patients, although it was originally designed to predict functional decline or death over a two-year period in community-dwelling elders. The higher sensitivity of the G8 screening tool has been reported in the literature. A previous independent report of 113 patients found greater sensitivity for G8 (85.7% *v* 57.1% for VES-13), although the AUC was not statistically different [33]. A recent systematic review [36] compared all available screening methods to CGA and reported a median sensitivity for VES-13 of 68% (range 39 to 88%), and median specificity of 78% (range 62 to 100%). The median sensitivity for G8 was higher at 87% (range 77 to 92%) with a median specificity of 61% (range 39 to 75%) [36].

VES-13 has been studied as a screening tool in oncology in a number of previous reports [26], [30], [31], [33], [37] and two of them concluded that VES-13 could be a useful preliminary screening tool with a sensitivity of 73% and 87% [28], [30]. However, similar analyses reported lower sensitivities ranging from 55 to 68.7% [26], [31], [33], [37]. These variations may result from differences in administration. In Luciani et al's study, VES-13 was administered by a physician and thus possibly over a longer time and in more detail [38] than in the present study or others, where VES-13 was administered predominantly by a nurse or CRA.

Additional tools, such as the Barber Questionnaire that was developed as a screening procedure for older adults in general practice [39] are available but results reported for older adults with breast cancer are disappointing [31]. Further geriatric tools have been proposed for screening purposes such as cancer specific geriatric assessment [40], the abbreviated (a)CGA [41], and the Groningen Frailty Index (GFI) [42]. However, overall, most of these instruments have only been presented in feasibility or pilot studies [25], and initial results suggest that they miss too many cases of vulnerable patients [26].

The false negative rate, undetected unfit patients, was lower with G8 than with VES-13: 23.5% *v* 31.3% patients respectively with MGA, and 13.5% *v* 21.5% with the modified MGA. The survival analyses demonstrated the strong prognostic role of the G8 score (HR  =  2.72, p<0.0001) along with male sex (HR 2.69, p<0.0001), poor ECOG status (HR 3.28, p<0.0001) and metastatic disease (HR 3.30, p<0.0001). The definition of the reference test was supported by the association observed between impaired MGA and poorer survival at one year in the exploratory survival analyses (HR 2.96, p = 0.0018). With specificity of 64.4% and NPV of 40.3%, the G8 tool still needs improvement. The somewhat low reproducibility observed for questions such as neuropsychological problems may be an issue for improvement, although part of the explanation may be the delay between the two tests that lasted up to one month.

Considering these results, the G8 questionnaire may be proposed to cancer patients over 70 years. Given its simplicity, this approach may allow physicians to discriminate fit from unfit patients. In this way, fit patients can benefit from standard treatment without extensive evaluation and efforts can be centered on unfit patients who need careful medical attention. For the latter, if resources are available, management should be multidisciplinary and based on appropriate geriatric assessment. If not, they should be offered at least cautious medical attention and case management when advanced practice nurses can be involved.

In summary, no current consensus exists to define target elderly population who may benefit from further medical attention before cancer treatment. Our definition which uses a threshold of one or two abnormal geriatric assessment questionnaires is probably the most reliable up to now and our exploratory survival analyses demonstrated the prognostic value of impaired MGA, but search for a refined reference test remains an issue

This study responds to a critical need for easy and quick-to-use screening tools to identify older patients requiring more detailed assessment and possible geriatric interventions. Screening has been recently encouraged for all patients that may benefit from full CGA in a recent SIOG/EUSOMA publication [43]. First and foremost, this study documents the high rate of older patients with geriatric impairments for whom oncologists lack clear directives and skills for practice and care. The G8 screening method recognizes the heterogeneity of the older patient population and provides oncologists with a useful, efficient tool to improve care.

## Supporting Information

S1 Appendix **The G8 questionnaire.**
(DOCX)

S2 Appendix **ONCODAGE abridged protocol (in English).**
(DOC)
