# Supplementary material for: Screening for Vulnerability in Older Cancer Patients: The ONCODAGE Prospective Multicenter Cohort Study
Source: PLoS One. 2014 Dec 11;9(12):e115060. doi: 10.1371/journal.pone.0115060 (PMC4263738; doi:10.1371/journal.pone.0115060)
Supplement: S1 Appendix — The G8 questionnaire. (DOCX) [file pone.0115060.s001.docx]

**Appendix S1 – G8 test in English**

| **MNA item** | **Items** | **Possible answers (score)** |
| --- | --- | --- |
| **A** | Has food intake declined over the past 3 months due to loss of appetite, digestive problems, chewing or swallowing difficulties? | 0 : severe decrease in food intake |
|  |  | 1 : moderate decrease in food intake |
|  |  | 2 : no decrease in food intake |
| **B** | Weight loss during the last 3 months | 0 : weight loss > 3 kg |
|  |  | 1 : does not know |
|  |  | 2 : weight loss between 1 and 3 kgs |
|  |  | 3 : no weight loss |
| **C** | Mobility | 0 : bed or chair bound |
|  |  | 1 : able to get out of bed/chair but does not go out |
|  |  | 2 : goes out |
| **E** | Neuropsychological problems | 0 : severe dementia or depression |
|  |  | 1 : mild dementia or depression |
|  |  | 2 : no psychological problems |
| **F** | Body Mass Index (BMI (weight in kg) / (height in m²) | 0 : BMI < 19 |
|  |  | 1 : BMI = 19 to BMI < 21 |
|  |  | 2 : BMI = 21 to BMI < 23 |
|  |  | 3 : BMI = 23 and > 23 |
| **H** | Takes more than 3 medications per day | 0 : yes |
|  |  | 1 : no |
| **P** | In comparison with other people of the same age, how does the patient consider his/her health status? | 0 : not as good |
|  |  | 0.5 : does not know |
|  |  | 1 : as good |
|  |  | 2 : better |
| **-** | Age | 0 : >85 |
|  |  | 1 : 80-85 |
|  |  | 2 : <80 |
|  | **TOTAL SCORE** | **0 – 17** |
