# Supplementary material for: Screening for Vulnerability in Older Cancer Patients: The ONCODAGE Prospective Multicenter Cohort Study
Source: PLoS One. 2014 Dec 11;9(12):e115060. doi: 10.1371/journal.pone.0115060 (PMC4263738; doi:10.1371/journal.pone.0115060)
Supplement: S2 Appendix — ONCODAGE abridged protocol (in English). (DOC) [file pone.0115060.s002.doc]

Appendix S2 – ONCODAGE study protocol (abridged translation from French)

| **Study title** | Validation of a geriatric screening tool in oncology |
| --- | --- |
| **Acronym** | **ONCODAGE** |
| **Coordinating centre** | Institut Bergonié, Centre de Lutte Contre le Cancer, Bordeaux. |
| **Coordinator** | Pr. Pierre Soubeyran |
| **Number of centres** | At least 15: the 15 Pilot Units of Coordination in Geriatric Oncology (*UPCOG*) along with health institutions members of the oncology network. |
| **Indication** | Patients over 70 years old with a cancer (colon, lung, ENT, breast, prostate, lymphoma), as part of the initial management. |
| **Trial rationale** | As patients age, risks for serious toxicity, or even lethality, during cancer treatment increase. A detailed assessment of the patient’s status is required.  Comprehensive geriatric assessment (CGA) is a method which has proved its usefulness but which is time-consuming and expensive to administer. A screening tool allowing the individualisation of patients who would benefit from a CGA is necessary.  In a previous regional prospective multicentre cohort (regional Ministry of Health-financed project, PHRC 2003), we studied patients over 70 years with cancers who were receiving first-line chemotherapy. This study enabled us to perform an exploratory study in order to develop a screening tool, the G8, which consists of a 7-item questionnaire from the Mini Nutritional Assessment (MNA) and an age indication. We applied the suggested screening tool to our population of 364 patients and this analysis provided encouraging results to be studied on a wider scale (Bellera et al., Ann Oncol, 2012). |
| **Expected effects** | With the use of this simple tool for screening, to be administered by nurses or oncologists, we consider that more elderly patients with cancer will be able to benefit from a CGA.  This tool will also enable us to rationalise the use of our geriatric assessment means and thus to focus their use on those who need it more, for more efficacy. |
| **Main objective** | Validate a new screening tool, the G8, to identify within a population of over 70-year-old patients with cancer those who need further geriatric assessment. |
| **Secondary objectives** | - **Validate the French version of the Vulnerable Elders Survey screening tool (VES-13).** - For each suggested screening tool, G8 and VES-13: - Assess the intrinsic qualities of the screening tool (internal consistency, reproducibility, and predictive value), - Assess the screening tool for specific populations (type of cancer, therapeutic strategy, stage of the disease). - Assess the number and type of suggested interventions according to the CGA - Compare both new suggested tools (VES 13 and G8). - Defining standards for tests and questionnaires used, for which no standards are available (G8, VES-13, MMSE, Timed up and Go, QLQ-C30) in a French population of elderly patients with cancer. |
| **Study design** | National multicentre prospective cohort study suggested to the 15 UPCOG and to health institutions members of oncology networks. |
| **Number of patients** | In order to ensure a minimum of 1500 assessable patients, we will recruit **1650 patients**. |
| **Inclusion criteria** | 1. 70-year-old patients or more,  2. Cancer proved histologically, irrespective of grade or stage,  3. The following tumour types: colon, lung, ENT, breast, and lymphoma,  4. As part of an initial therapeutic management, whether it is medical (chemotherapy, hormone therapy, targeted treatment), surgical, or radiotherapeutic,  5. Management by one of the 15 UPCOG or teams associated to the project,  6. Patients who received the information leaflet and signed the informed consent form,  7. Patients registered with a social security scheme. |
| **Non-inclusion criteria** | 1. Persons deprived of freedom or under tutelage,  2. Presence of a psychological, familial, social, or geographic condition which might disturb the right course of the study. |
| **Course of the study** | **Oncological assessment consultation** *(oncologist and nurse)*   - Information leaflet and informed consent, - Assessment of eligibility criteria, - Standard clinical examination (oncologist), - G8 questionnaire (oncologist), - VES-13 self-questionnaire (nurse), - Organisation of appointments for oncological assessment (pre-therapeutic evaluation, extended assessment), and geriatric oncologic assessment.   **Oncological assessment**  The oncological assessment is the extended and standard pre-therapeutic assessments usually performed for patients. Clinical and paraclinical data will be collected for the study.  **Geriatric oncologic assessment** *(geriatrician and geriatric assessment nurse for UPCOG)*  Before starting the oncological treatment and before setting up specific management, within two weeks at least after the oncological assessment consultation.  The nurse (IEG) will have to:   - Filling ADL, IADL, MNA questionnaires, along with the socio-cultural assessment score, - Performing the MMS and Timed Get up and Go tests, - Filling GDS-15 and QLQ- C30 self-questionnaires.   The geriatrician will then receive the patient for an oncological geriatrics consultation and will be in charge of the CIRS-G questionnaire, the synthesis of the self-questionnaires, the oncological geriatrics assessment, and of the clinical file. The doctor will try to recognise the patient for who the setting of a personalised programme of geriatric interventions is required.  **It is essential for the geriatrician not to have access to the G8 nor to the VES-13.**  **Follow-up**  Patients will be followed over 5 years. Data collection will be done at 1 and 5 years in order to collect data on vital status, disease progression, lifestyle (ambulatory, institutionalised). |
| **Summary of the study course** | **Step 1: Inclusion (J0)**  (Medical oncologists, surgeons, radiotherapists...)  _ Information leaflet  _ Informed consent  _ Inclusion and non-inclusion criteria  _ Inclusion form  **Step 1: Initial oncologic consultation (J0)**  (Medical oncologists, surgeons, radiotherapists...)  _ Booking an appointment for the geriatrics consultation  _ General information on the patient  _ Information on cancer  _ G8 questionnaire  _ VES-13 self-questionnaire  **Step 2: Oncological extended assessment (J0-J30)**  _ Biological data  **Step 3: Geriatrics consultation (J1-J30)**  (Geriatrician and nurse)  _ Geriatric assessment:  _ MMSE  _ ADL, IADL  _ GDS-15  _ QLQ-C30, sociocultural assessment  _ Timed Get up and Go  _ MNA  _ CIRS-G  _ Geriatrician’s recommendations  VES-13 again for 60 patients/centre in three centres:  Bordeaux, Lille and Lyons  **Step 4: Follow-up at 1 and 5 years**  Follow-up data collection (vital status and lifestyle) |
| **Assessment criteria** | **Principal assessment criteria**  G8 assessment will be performed using as a gold-standard tool the complete CGA based on the following tests, questionnaires, and scales: CIRS-G, ADL and IADL scales Timed Get up and Go test, MNA, MMS, and GDS-15.  Especially, we will consider a patient as being at risk or requiring a complete CGA if we observe at least one of the following conditions:   - CIRS-G: at least one grade ≥ 3 comorbidity (except treated cancer), - ADL scale: a score below or equal to 5, - IADL scale: a score below or equal to 7, - Timed Get up and Go test: time strictly over to 20 seconds, - MNA: a score below or equal to 23.5, - MMSE: a score below or equal to 23, - GDS-15: a score over or equal to 6.   We will assess, for various thresholds of the assessed tool, the performances of the new screening tool compared to the reference one, thanks to the following parameters:   - Sensitivity and specificity, - Youden’s index (J), - The predictive values (positive or negative), - The diagnostic likelihood ratios (positive or negative), - The area under the ROC curve.   **Secondary assessment criteria**   - The validation of the French version of the VES-13 screening tool will be based on the same validation criteria than for G8 tool. - The assessment of the intrinsic qualities of G8 and VES-13 tools will be based on the accuracy (internal consistency, reproducibility) and validation assessment. - The assessment of screening tools in various clinical situations will be based on the estimation of sensitivity, specificity, and of predictive values of each screening tool according to: - the type of cancer, - the therapeutic strategy (locoregional, and locoregional and adjuvant treatment), - the stage of the disease (local, metastasis). - We will evaluate the number of interventions according to the CGA (change of the initial treatment irrespective of its nature, interventions of other specialists in other fields). |
| **Hypotheses and statistical analyses** | **Hypotheses and required number of patients**  The objective is to maximise the sensitivity of the new screening tool and to obtain an estimation with a satisfying accuracy. Our exploratory study suggests (section 8) a sensitivity of about 90%, when we use as gold standard the presence of an abnormal score for at least one questionnaire, and a prevalence of 50% of patients with at least one abnormal questionnaire.  The inclusion of 1500 patients would help estimating the sensitivity on a 750-patient sample with abnormal CGA, with an accuracy of 2.4% for an expected sensitivity of 90% (*i.e.* 95% CI of 87.6% to 92.4%). In order to ensure a minimum of 1500 assessable patients, we will recruit **1650 patients**.  **Statistical analyses**  The population of the study will be described broadly according to its clinical and sociodemographic characteristics in terms of size and ratio for the qualitative variables and in terms of distribution for the quantitative ones.  The following parameters will be estimated:   - The sensitivity, the specificity, and the predictive values will be estimated. 95% CIs will be reported (binomial distribution). - The ROC curve representing graphically the sensitivity according to the (1 -specificity) for various thresholds of the assessed test will be represented graphically and help determine the area under the curve (AUC). - Internal consistency will be assessed according to Cronbach’s alpha coefficient. A 95% CI will be reported. - Reproducibility will be assessed by the intraclass correlation coefficient for quantitative variables. For qualitative variables, reproducibility will be assessed by Cohen’s kappa. - Scores between acknowledged subgroups will be compared thanks to a variance analysis. - Both G8 and VES-13 questionnaires will be compared thanks to their respective area under the curve. - We will assess whether using both screening tools together (G8 and VES-13) helps obtaining a more effective screening tool compared to the sole use of one questionnaire. |

**3.3 ENDPOINTS (PROTOCOLE REMINDER)**

**3.3.1 Primary endpoints**

G8 assessment will be performed using as a gold-standard tool the CGA questionnaires, tests, and scales: CIRS-G, ADL and IADL scales, Timed Get up and Go test, MNA, MMSE, and GS-15.

Especially, we will consider a patient as being at risk or requiring a complete CGA if we observe at least one of the following conditions:

CIRS-G: at least one grade ≥ 3 comorbidity (except for cancer),

ADL scale: a score below or equal to 5,

IADL scale: a score below or equal to 7,

Timed Get up and Go test: time strictly over to 20 seconds,

MNA: a score below or equal to 23.5,

MMSE: a score below or equal to 23,

GDS-15: a score over or equal to 6.

**These assessments will be performed blindly from the G8 and VES-13 answers.**

We will assess, for various thresholds of the assessed tool, the performances of the new screening tool compared to the reference one, thanks to the following parameters:

**The sensitivity (Se)**, defined by the proportion of patients registered as “requiring an CGA” with the screening tool, among patients having at least one abnormal CGA element;

**The specificity (Sp)**, defined by the proportion of patients registered as “not requiring a CGA” with the screening tool, among patients “having no abnormal CGA element”;

**Youden’s index (J)**, synthesizing the sensitivity and the specificity: J = Se + Sp – 1.

**The positive predictive value (PPV)**, defined by the proportion of patients “having at least one abnormal CGA element”, among patients registered as “requiring an CGA” with the screening tool.

**The negative predictive value (NPV)**, defined by the proportion of patients registered as “having no abnormal CGA element”, among patients “not requiring an CGA” with the screening tool;

**The positive diagnostic likelihood ratios (DLR+)**, defined by the ratio of positive tests in patients with the disease (true positive) out of positive tests in disease-free patients (FN of false positive).

**The negative diagnostic likelihood ratio (DLR-)**, defined by the ratio of negative tests in patients with the disease (false negative) out of negative tests in disease-free patients (true positive).

The area under the ROC curve will help determining the overall efficacy of the assessed test and determining the threshold value to increase simultaneously sensitivity and specificity of the new screening tool, or to maximise sensitivity while keeping a satisfying specificity. We will try to maximise sensitivity in order to decrease the G8 false negative rates, which would not thus benefit from the best management later.

**3.3.2 Secondary endpoints**

3.3.2.1 Validation of the French version of the VES-13 screening tool

We will use the same validation criteria than for the G8 tool (see previous section): sensitivity, specificity, Youden’s index, predictive values, diagnostic likelihood ratios.

3.3.2.2 Assessment of G8 and VES-13’s intrinsic qualities

We will assess the accuracy and the validity of each screening tool, G8 and VES-13 (Falissard 2001).

**Accuracy assessment:**

**The internal consistency** of each questionnaire will be assessed from answers to every item and according to Cronbach’s alpha coefficient,

The reproducibility will be assessed

- Inter-judges mistakes for G8: comparing the questionnaire filled by the oncologist and the same questions for G8 filled by the nurse (IGE) for each item and the overall score,

- Test-retest for VES-13: comparing the self-questionnaire filled by the patient during the oncology consultation to the questionnaire filled during the geriatrics consultation. This study will be conducted in three centres only: Bordeaux, Lille, and Lyons (60 patients per centre. See chapter 16.1.2 for a justification of the number of patients).

**Assessment of the discriminating validity:**

A comparison of the score obtained to G8 and VES-13 between different groups according to the expected risk level of geriatric disorder will be performed to assess whether these questionnaires help discriminating populations for which we expect score differences (for instance, patients over 85 *vs.* patients under 85, palliative *vs.* non-palliative…).

**Assessment of the predictive validity:**

The G8 and VES-13 predictive validity will be assessed at 1 and 5 years according to the following events: mortality, disease progression, and institutionalisation.

3.3.2.3 Assessment of screening tools in various clinical situations

We will assess sensitivity, specificity, and predictive values for each screening tool, G8 and VES-13:

The type of cancer (colon, lung, ENT, breast, and lymphoma),

The therapeutic strategy (according to three groups: locoregional treatment, locoregional and adjuvant treatment, general treatment),

The stage of the disease (local, metastasis)

3.3.2.4 Assessment of geriatrics interventions

We will assess the number of interventions according to the CGA (change of initial treatment irrespective of its nature, interventions of other specialists from other fields (psychologists, psychiatrists…), nutritional intervention, kinesitherapy, and social intervention for instance), in the general population, and by type of cancer.

We will also assess each screening tool using as a reference tool the indication for geriatric intervention. We will study sensitivity, specificity, and predictive values for G8 for screening anomalies requiring a geriatric intervention.

**3.4 STATISTICAL FACTORS**

**3.4.1 Hypotheses and required number of patients**

3.4.1.1 Main analysis

For a screening tool, sensitivity and specificity are assessed jointly, although we tend to favour sensitivity as it could be damageable not to detect patients requiring a CGA. The objective then is to maximise the sensitivity of the new screening tool.

Our exploratory study suggests (Chapter 9):

a sensitivity of about 90%, when we use as gold standard the presence of an abnormal score for at least one questionnaire,

a prevalence of 50% of patients with at least one abnormal questionnaire.

Supposing that the real sensitivity for our G8 screening tool is 90%, we estimate that the inclusion of 750 patients with at least one abnormal questionnaire would be enough to estimate the sensitivity with a sufficient accuracy (2.4%) and to obtain a 95% CI between 88% and 92%. Supposing that 50% of our population will have at least one abnormal questionnaire, **1500 assessable patients** will be needed.

A patient is said to be assessable if CGA and G8 data are complete. In our exploratory study, only 309 patients out of 364 had all the necessary questionnaires for CGA (85%). In order to ensure a minimum of 1500 assessable patients, we will recruit 1650 patients.

If we want to estimate sensitivity according to the type of cancer, we will obtain an accuracy of +/- 6% for a sample of 200 patients (95% CI: 84% to 96%), and of about 5% for a sample of 400 patients (95% CI: 86% to 94%).

3.4.1.2 Estimation of the VES-13 reproducibility

The test-retest reproducibility study for the VES-13 questionnaire will be performed on a sample of patients from the Oncodage study. Pearson’s reproducibility coefficient after one month for the English VES-13 in a population of patients over 70 treated by anti-androgens for a prostatic cancer (Mohile et al., 2007) was 0.92 overall (respectively 0.52, 0.60, and 0.70 for the subsets concerning health perception, physical and functional disabilities) but data for the intraclass correlation coefficient were not found in the literature for the VES-13 test-retest assessment.

For two measures, the inclusion of 160 patients would provide an estimation for the intraclass correlation coefficient of 0.6 with an accuracy corresponding to a 95% CI of 0.2 width, *i.e.* 95% CI = [0,5-0,7] (Bonett, 2002).The accuracy obtained will be even better if the obtained coefficient is higher.

In order to be sure of getting 160 patients assessed by the VES-13 at both times (oncologic consultation and geriatric assessment), it has been decided to included 180 patients, corresponding to the first 60 patients who will be included in the three participating centres (Bordeaux, Lille, Lyons).

**3.4.2 Study population**

The main analysis, the validation of the screening tool, will be conducted on every patient included in the study.

Secondly, the new screening tool will also be assessed according to:

- the type of cancer,
- the stage of the cancer (local/metastasis),
- the therapeutic strategy (according to three groups: locoregional treatment, locoregional and adjuvant treatment, general treatment).

**3.4.3 Statistical analysis**

The population of the study will be described broadly according to its clinical and sociodemographic characteristics in terms of size and ratio for the qualitative variables and in terms of distribution for the quantitative ones.

Missing data will be reported (by item and by questionnaire) thanks to frequency and percentage. The reasons for these missing data for any test will be described.

The following parameters will be estimated:

- **The sensitivity** will be estimated with the proportion of patients registered as “requiring a geriatric intervention” by the new tool, among the patients having at least one abnormal CGA element. A 95% CI will be reported (binomial distribution).
- **The specificity** will be estimated with the proportion of patients registered as “not requiring a geriatric intervention” by the new tool, among the patients having no abnormal CGA element.

A 95% CI will be reported (binomial distribution).

- **The positive predictive value** will be estimated with the proportion of patients having at least one abnormal CGA element, among the patients registered as “requiring a geriatric intervention” by the new tool. A 95% CI will be reported.
- **The negative predictive value** will be estimated with the proportion of patients having no abnormal CGA element, among the patients registered as “not requiring a geriatric intervention” by the new tool. A 95% CI will be reported (binomial distribution).
- **The ROC curve** representing graphically the sensitivity according to the (1 -specificity) for various thresholds of the assessed test will be represented graphically and help determine the area under the curve (AUC).
- **The internal consistency** will be assessed according to Cronbach’s alpha coefficient (Armitage & Berry, 2002).A 95% CI will be reported.
- **The reproducibility** will be assessed by the intraclass correlation coefficient for quantitative variables, with a 95% CI.
- **Scores between acknowledged subgroups** will be compared thanks to a variance analysis.
- **The area under the ROC curve** for G8 and VES-13 will be compared (Hanley et al, 1983).
- **We will assess whether using both screening tools together** (G8 and VES-13) helps obtaining a more effective screening tool compared to the sole use of one questionnaire (Macaskill et al., 2002).
- **Biological data** will be described thanks to descriptive statistics for overall population and then by localisation. We will thus assess whether inclusion of these variables (individually or simultaneously) in the new screening tool participates in the improvement of the tool’s achievements. We will study the screening tool’s sensitivity and specificity when it takes into account these biological data.
- **For the prognostic analysis** (predictive validity study), delays will be calculated between the origin date (inclusion in the study) and the event date (death, progression, or institutionalisation). Survival curves will be represented graphically with Kaplan-Meier’s method (Kaplan et al. 1958). Median times for survival will be reported with a 95% CI. Patients will be compared according to the categories defined by the G8 and VES-13 thresholds determined by the analysis of the corresponding objectives of our study, thanks to a Log-Rank test, if application conditions are respected. A Cox regression model (Cox, 1972) will enable us to determine the relative risks (RR) (and their CI) associated to VES-13 and G8 initial results for the occurrence of various events of interest (death and institutionalisation), taking into account the durations. A stratified analysis on the various prognostic factors (independent from CGA) will be performed: such as type of cancer, or initial grade. The analysis of the “institutionalisation” risk will be only on the patients not institutionalised at the inclusion (population at risk).
- **Regarding the particular case of** **MMSE,** secondary analyses for robustness will be performed using different thresholds to define an altered MMSE, taking into account the score distribution according to the education level and the age.

All the analyses will be performed with the SAS 9.1. software, with a type I error of 5%.

**4. KEY DATES**

26/03/2008 Initial ethics application

14/05/2008 Scientific board meeting (am) Investigators meeting (pm)

27/05/2008 AFSSAPS Authorisation

05/08/2008 Beginning of inclusions

08/09/2009 Scientific board meeting

16/10/2009 IDMC

08/03/2010 End of inclusions

22/02/2010 to 14/09/2010 Monitoring onsite (19 sites / 528 files / 32% of included patients)

15/09/2010 to 12/10/2010 Preliminary analysis

13/10/2010 Preliminary results presentation (INCa + scientific board)

01/11/2010 to 31/01/2011 Finalisation of analyses (except for the follow-up analysis)

**31/01/2011 Final report n°1**

30/04/2011 Follow-up data at 1 year available for all patients

01/05/2011 to 30/05/2011 Follow-up data analyses at 1 year

30/04/2015 Follow-up data at 5 years available for all patients

01/05/2015 to 30/05/2015 Follow-up data analyses at 5 years
